# Transcatheter Valve-in-Valve Procedures for Bioprosthetic Valve Dysfunction in Patients With Rheumatic vs. Non-Rheumatic Valvular Heart Disease

**DOI:** 10.3389/fcvm.2021.694339

**Published:** 2021-08-04

**Authors:** Mariana Pezzute Lopes, Vitor Emer Egypto Rosa, José Honório Palma, Marcelo Luiz Campos Vieira, Joao Ricardo Cordeiro Fernandes, Antonio de Santis, Guilherme Sobreira Spina, Rafael de Jesus Fonseca, Mauricio F. de Sá Marchi, Alexandre Abizaid, Fábio Sândoli de Brito, Flavio Tarasoutchi, Roney Orismar Sampaio, Henrique Barbosa Ribeiro

**Affiliations:** Heart Institute (InCor) Clinical Hospital, University of Sáo Paulo, Sáo Paulo, Brazil

**Keywords:** heart valve prosthesis, rheumatic heart disease, bioprosthesis, mitral valve, aortic valve, transcatheter valve-in-valve, transapical access, transeptal access

## Abstract

**Background:** Bioprosthetic heart valve has limited durability and lower long-term performance especially in rheumatic heart disease (RHD) patients that are often subject to multiple redo operations. Minimally invasive procedures, such as transcatheter valve-in-valve (ViV) implantation, may offer an attractive alternative, although data is lacking. The aim of this study was to evaluate the baseline characteristics and clinical outcomes in rheumatic vs. non-rheumatic patients undergoing ViV procedures for severe bioprosthetic valve dysfunction.

**Methods:** Single center, prospective study, including consecutive patients undergoing transcatheter ViV implantation in aortic, mitral and tricuspid position, from May 2015 to September 2020. RHD was defined according to clinical history, previous echocardiographic and surgical findings.

**Results:** Among 106 patients included, 69 had rheumatic etiology and 37 were non-rheumatic. Rheumatic patients had higher incidence of female sex (73.9 vs. 43.2%, respectively; *p* = 0.004), atrial fibrillation (82.6 vs. 45.9%, respectively; *p* < 0.001), and 2 or more prior surgeries (68.1 vs. 32.4%, respectively; *p* = 0.001). Although, device success was similar between groups (75.4 vs. 89.2% in rheumatic vs. non-rheumatic, respectively; *p* = 0.148), there was a trend toward higher 30-day mortality rates in the rheumatic patients (21.7 vs. 5.4%, respectively; *p* = 0.057). Still, at median follow-up of 20.7 [5.1–30.4] months, cumulative mortality was similar between both groups (*p* = 0.779).

**Conclusion:** Transcatheter ViV implantation is an acceptable alternative to redo operations in the treatment of patients with RHD and severe bioprosthetic valve dysfunction. Despite similar device success rates, rheumatic patients present higher 30-day mortality rates with good mid-term clinical outcomes. Future studies with a larger number of patients and follow-up are still warranted, to firmly conclude on the role transcatheter ViV procedures in the RHD population.

## Introduction

Rheumatic heart disease (RHD) is a prevalent condition, especially in low- and middle-income countries. The Global Burden of Disease study estimated 10.5 million disability-adjusted life years and 319,499 deaths in 2015 due to RHD (1). In Brazil, the estimated annual incidence reaches 30,000 new cases per year, leading to a high cardiac mortality of −8% (2–4). Of note, RHD population has singular characteristics comparing to other etiologies of valvular heart disease. In general, RHD patients are operated at a younger age and undergo several open-heart surgeries during their lifetime, due to structural valve degeneration (SVD) which occurs earlier in these patients who are first-time operated at a very young age. The current standard treatment for degenerated bioprosthesis involves redo open-heart surgery. However, for many RHD patients, with multiple co-morbidities, such as left ventricular dysfunction, pulmonary artery hypertension and prior surgical procedures, a conventional reoperation poses additional risks.

Transcatheter valve interventions have been established as an alternative to conventional surgical interventions in recent years, initially for patients with severe aortic stenosis of various surgical risk profiles. More recently, this procedure has also been evaluated in patients with bioprosthetic valve failure [valve-in-valve (VIV)] in aortic, mitral and tricuspid positions (4–6), with acceptable clinical outcomes in the short- and long-term follow-up (1, 7–13). The aim of this study was therefore to evaluate the clinical characteristics and outcomes in rheumatic vs. non-rheumatic patients undergoing ViV procedures for severe bioprosthetic valve dysfunction.

## Materials and Methods

### Study Population

Single center prospective study including consecutive patients undergoing transcatheter ViV implantation, from May 2015 to September 2020. All cases were thoroughly discussed by the institutional Heart Valve Team, and patients were elected for transcatheter approach based on (i) preoperative risk assessment (STS ≥ 8.0% or EuroSCORE II ≥ 6.0%), (ii) presence of comorbidities, (iii) number of previous surgical interventions, (iiii) frailty and other clinical conditions.

Rheumatic etiology of the native valve disease was considered according to the referred clinical history, previous echocardiographic and surgical findings. Exclusion criteria were: (i) active endocarditis, (ii) presence of prosthetic valve thrombosis or thrombus in the left ventricle, and (iii) paravalvular regurgitation. The occurrence of thrombus in the left atrial appendage was considered a relative contraindication and evaluated individually. The study protocol was reviewed and approved by the local institutional ethics committee. All patients provided written informed consent for the procedures.

### Preoperative Planning

All patients underwent transthoracic echocardiographic analysis and, whenever necessary, a tridimensional transesophageal was performed. A cardiac gated thoracic computed tomography was also performed in order to obtain adequate measurement of the degenerated bioprosthetic valve's internal diameter (i.e. the True ID), in addition to other important measurements as previously described (14–16). Measurements were performed under multi-planar reconstruction, using OsiriX® Platform, and 3D reconstruction was performed to calculate the ideal fluoroscopic angulation for valve deployment. Coronary angiography was performed routinely, and precluded, at the Heart Team's discretion, if renal function was critical.

### Valve-in-Valve Procedure

Transcatheter procedures were performed routinely in hybrid operating room, according to standard techniques. Procedures were guided by transesophageal echocardiography and fluoroscopy using prosthesis metallic rings to position the transcatheter valve.

The self-expandable CoreValve and Evolut R (Medtronic, Minneapolis, MN), the balloon-expandable Sapien XT and Sapien 3 (Edwards Lifesciences, Irvine, CA) and the balloon-expandable Inovare (Braile Biomedica, Sao Jose do Rio Preto, SP) prostheses were used, at the discretion of the operator.

### Data Collection and Analysis

Pre and postoperative data were prospectively collected and entered into our institutional database. Data regarding 30-days outcomes and follow-up were retrospectively analyzed according to the Mitral Valve Academic Research Consortium (MVARC-2) and Aortic Valve Academic Research Consortium-2 (VARC-2) (17, 18). Continuous variables were presented as mean ± SD or median (interquartile range). Categorical variables were presented as percentages. Kolmogorov-Smirnov test was used to test normality of the variable. *T* test or Mann-Whitney test was applied for continuous variables, and Fisher exact test or χ^2^ test was applied for categorical variables, as appropriate. Log transformation was applied to normalize the distribution of STS score, creatinine, left ventricular end-diastolic diameter and left ventricular end-diastolic volume. Age, left ventricular end-systolic diameter, left ventricular end-systolic volume and left ventricular ejection fraction were analyzed using Mann-Whitney test. A logistic regression analysis was used to evaluate the predictors of device success. For mitral procedures, a MVARC modified criteria of device success was used as follow: absence of (i) procedural death, (ii) malposition/embolization/migration, (iii) second transcatheter heart valve, (iv) left ventricular outflow tract obstruction and (v) stroke. (19). For the aortic procedures, the VARC-2 criteria was used: (i) absence of procedural mortality, (ii) correct positioning of a single prosthetic heart valve into the proper anatomical location, (iii) no prosthesis–patient mismatch, (iv) mean aortic valve gradient <20 mmHg and (v) no moderate or severe prosthetic valve regurgitation (18). For the tricuspid ViV procedures, the following criteria was used: (i) absence of reintervention, endocarditis or valve thrombus, (ii) absence of moderate or severe regurgitation and (iii) absence of mean gradient ≥10 mmHg (4). Time-to-event analyses were performed using Kaplan-Meier estimates and groups were compared using log-rank test. All analyses were conducted using the statistical package SPSS, version 20 (IBM, Armonk, NY).

## Results

### Patient Characteristics

The main baseline clinical, laboratory and echocardiographic data are shown in Table 1. Among 106 patients included, 65.1% (*n* = 69) had rheumatic etiology and 34.9% (*n* = 37) had non-rheumatic etiology. The main non-rheumatic etiologies were mitral valve prolapse (32.4%), degenerative aortic stenosis (21.6%), congenital valve disease (16.2%) and post-endocarditis (13.5%). There were no demographic differences between rheumatic and non-rheumatic patients, except for more female sex (73.9 vs. 43.2%, respectively; *p* = 0.004) and atrial fibrillation (82.6 vs. 45.9%, respectively; *p* < 0.001) in rheumatic patients. There was also no difference regarding the median number of previous surgeries between rheumatic and non-rheumatic patients, nonetheless when stratified by the number of procedures, rheumatic patients had a greater number of ≥2 previous surgeries than non-rheumatic (68.1 vs. 32.4%, respectively; *p* = 0.001). In addition, rheumatic patients had smaller left ventricular end-diastolic diameter than non-rheumatic patients (50.0 ± 6.6 vs. 54.6 ±10.7 mm, respectively; *p* = 0.020), and higher pulmonary artery systolic pressure (62.3 ± 19.7 vs. 52.7 ± 17.1 mmHg, respectively; *p* = 0.029). No other echocardiographic differences were seen between groups. The mechanisms for surgical biosprosthetic failure were similar between the groups, as detailed in Table 1.

**Table 1:** Baseline clinical, laboratory and echocardiographic data of the study population.

	**Rheumatic (*N* = 69)**	**Non-rheumatic(*N* = 37)**	***P* value**
**Clinical data**			
Age, years	63 ± 10	59 ±22	0.684
Body surface area, m^2^	1.61 ± 0.14	1.66 ± 0.19	0.170
Female sex	51 (73.9)	16 (43.2)	0.004
Diabetes	10 (14.5)	7 (18.9)	0.753
Hypertension	36 (52.2)	22 (59.5)	0.608
Poor mobility	16 (23.2)	6 (16.2)	0.554
Atrial fibrillation	57 (82.6)	17 (45.9)	<0.001
Coronary artery disease	14 (20.3)	8 (21.6)	1.000
Previous heart failure hospitalization	13 (18.8)	8 (21.6)	0.931
Number of previous surgeries	2 (3–1)	1 (2–1)	0.103
Total of previous surgeries			0.061
1	20 (29.0)	22 (59.5)	
2	22 (31.9)	4 (10.8)	
3	20 (29.0)	6 (16.2)	
4	3 (4.3)	2 (5.4)	
5	2 (2.9)	2 (5.4)	
6	2 (2.9)	1 (2.7)	
≥2 previous surgeries	47 (68.1)	12 (32.4)	**0.001**
STS-PROM score, %	7.93 ±5.47	6.61 ±5.07	0.174
**Symptoms**			
NYHA class			0.547
I	4 (5.8)	6 (16.2)	
II	18 (26.1)	7 (18.9)	
III	30 (43.5)	14 (37.8)	
IV	17 (24.6)	10 (27.0)	
Angina	9 (13.0)	3 (8.1)	0.535
**Laboratory**			
Hemoglobin, mg/dl	11.98 ± 1.94	12.41± 2.14	0.315
Creatinine, mg/dl	1.24± 0.55	1.24 ± 0.47	0.935
**Baseline echocardiography**			
LVED diameter, mm	50.03 ± 6.60	54.68 ±10.72	**0.020**
LVES diameter, mm	33.82 ± 6.18	38.32 ± 11.52	0.147
LVED volume, ml	117.47 ± 41.55	137.80 ± 71.32	0.254
LVES volume, ml	51.02 ± 36.50	63.53 ± 55.15	0.226
LVEF, %	59.8 ±9.6	55.0 ± 11.3	0.265
PSAP, mmHg	62.3 ± 19.7	52.7 ± 17.1	**0.029**
**Valve Failing Mechanism**			
Aortic	**(** ***N*** **=** **6)**	**(** ***N*** **=** **10)**	0.271
Stenosis	2 (33.3)	6 (60)	
Regurgitation	3 (50)	4 (40)	
Mixed	1 (16.7)	-	
Mitral	**(** ***N*** **=** **59)**	**(** ***N*** **=** **17)**	0.170
Stenosis	27 (45.8)	7 (41.2)	
Regurgitation	24 (40.7)	10 (58.8)	
Mixed	4 (6.8)	-	

*Values are n (%), mean (±SD) or median [IQR]. NYHA, New York Heart Association; eGFR, estimated glomerular filtration; STS-PROM, Society of Thoracic Surgeons predicted risk of mortality; LVED, Left ventricular end-diastolic; LVES, Left ventricular end-systolic; LVEF, left ventricular ejection fraction; PSAP, pulmonary systolic arterial pressure. The bold values refer p-values <0,05 (statistically significant)*.

### Procedural Data

**Figure 1:**
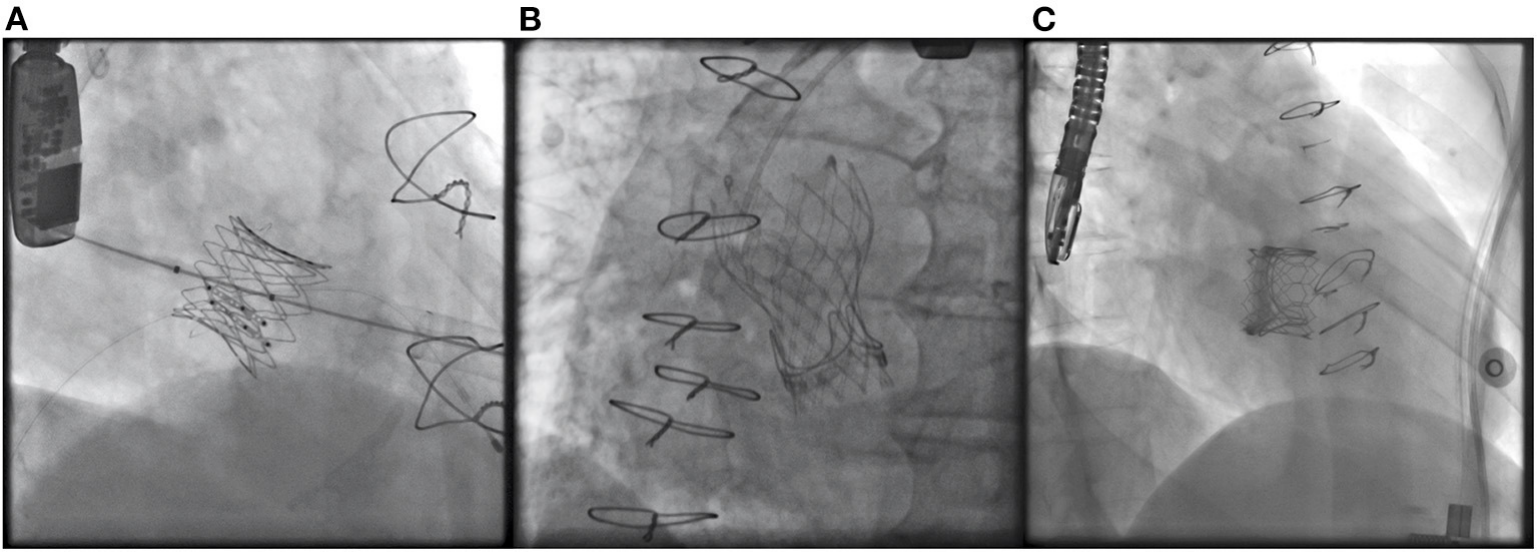
Case examples of a **(A)** balloon expandable Inovare bioprosthesis implanted in the tricuspid position. **(B)** Self-expandable Evolut R bioprosthesis implanted in the aortic position. **(C)** Balloon expandable Sapien 3 valve implanted in the mitral position.

The main baseline procedural data are shown in Table 2. We found no difference between rheumatic vs. non-rheumatic patients regarding time between bioprosthesis implantation and ViV procedure (12 [9–16] vs. 12 [7–15] years, respectively; *p* = 0.871). Rheumatic patients underwent more mitral valve-in-valve procedure than non-rheumatic (81.2 vs. 45.9%, respectively; *p* = 0.025), while non-rheumatic patients underwent more aortic and tricuspid ViV procedures, both in 27.0% of patients, respectively. Examples of ViV procedures with the different transcatheter valves in the various positions are shown in Figure 1. Most of RHD patients underwent transapical access (92.6%) while non-rheumatic patients had transapical access in 70.3%, jugular access in 13.5%, and both transfemoral and transeptal in 8.1% of patients, respectively (*p* = 0.008). Despite the differences in procedural characteristics, device success rate was similar between the groups (75.4 vs. 89.2% in rheumatic vs. non-rheumatic, respectively; *p* = 0.148). There was no significant predictor of device success in the univariable analysis (Supplementary Table 1).

**Table 2:** Procedural data of the study population.

**Procedure data**	**Rheumatic (*N* = 69)**	**Non-rheumatic(*N* = 37)**	***P* value**
Device success*	52 (75.4)	33 (89.2)	0.148
Pre-dilatation	1 (1.4)	6 (16.2)	**0.007**
Post-dilatation	23 (33.3)	8 (22.2)	0.337
Access			**0.008**
Transapical	63 (92.6)	26 (70.3)	
Jugular	1 (1.4)	5 (13.5)	
Transfemoral	2 (2.9)	3 (8.1)	
Transeptal	2 (2.9)	3 (8.1)	
Position			**0.025**
Mitral	56 (81.2)	17 (45.9)	
Aortic	6 (8.7)	10 (27.0)	
Tricuspid	0	10 (27.0)	
Multiple	7 (10.1)	0	
Length of in-hospital stay, days	18 (30–10.5)	16 (24.5–8)	0.425

*Values are mean ± SD, median (interquartile range), or n (%)*.

* *According to the modified Mitral Valve Academic Research Consortium (MVARC-2) without hemodynamic criteria (SIMONATO) and the Aortic Valve Academic Research Consortium-2 (VARC-2) (9, 10, 19). The bold values refer p-values <0,05 (statistically significant)*.

### Clinical Outcomes and Follow-Up

**Figure 2:**
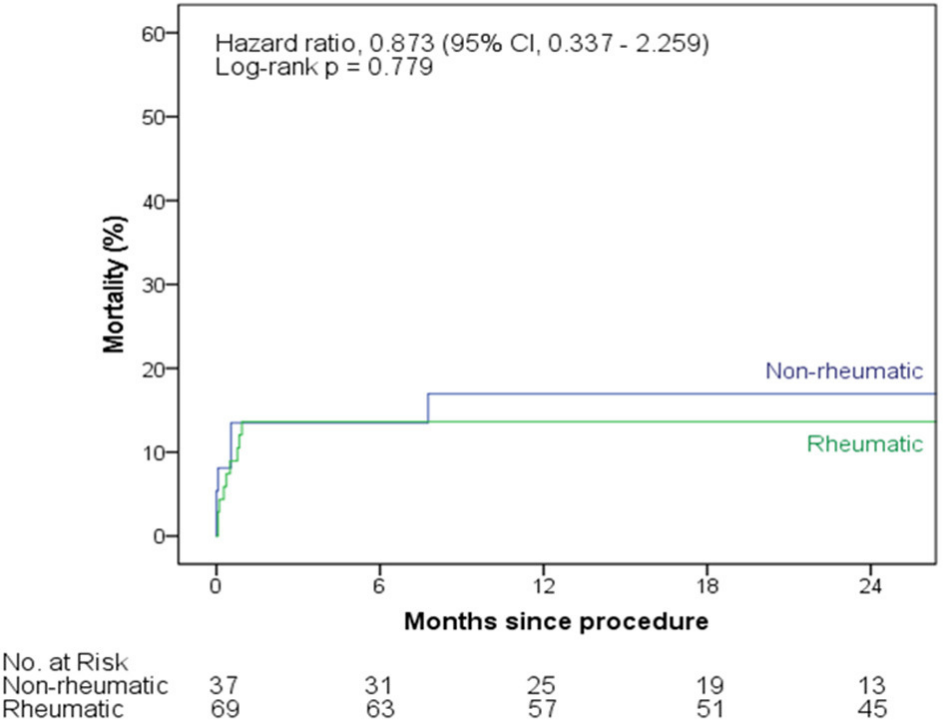
Kaplan-Meier survival curves at 2-year follow-up, according to rheumatic vs. non-rheumatic valve disease.

The main procedural outcomes are shown in Table 3. There were no significant differences between groups. The most frequent post-procedural complications were major bleeding and the need for packed red blood cells transfusion in 10.4 and 14.2% of patients, respectively. Also, up to 15.1% of the patients presented acute kidney injury, needing dialysis in 2.8% of them, and valve-related dysfunction requiring valve surgery occurred in 5 patients, being 4 (5.8%) and 1 (2.7%) in rheumatic vs. non-rheumatic patients respectively (*p* = 0.656). At 30-days, there was a trend toward higher mortality rates in rheumatic vs. non-rheumatic patients (21.7 vs. 5.4%, respectively; *p* = 0.057). In the univariable analysis (Supplementary Table 2) rheumatic etiology was significantly associated with 30-day mortality (OR 4.861, 95% CI 1.047–22.573, *p* = 0.044). At a median follow-up of 20.7 [5.1–30.4] months, rheumatic etiology was not associated with mid-term mortality (HR 0.873, 95% CI 0.337–2.259, *p* = 0.779; Figure 2). Post-procedure echocardiographic data, as prosthesis mismatch and prosthetic paravalvular leak (PVL), are demonstrated in Supplementary Table 3.

**Table 3:** 30-day clinical outcomes.

**Outcomes**	**Rheumatic (*N* = 69)**	**Non-rheumatic(*N* = 37)**	***P* value**
Procedure mortality	1 (1.4)	1 (2.7)	1.000
In-hospital myocardial infarction	4 (5.8)	1 (2.7)	0.656
Major vascular complication	3 (4.3)	1 (2.7)	1.000
Major bleeding	7 (10.1)	4 (10.8)	1.000
Red blood cells transfusion	11 (15.9)	4 (10.8)	0.667
Acute kidney injury	10 (14.5)	6 (16.2)	1.000
Sepsis	19 (27.5)	6 (16.2)	0.285
Valve-related dysfunction requiring second valve implantation	0	1 (2.7)	0.349
Valve-related dysfunction requiring valve surgery	4 (5.8)	1 (2.7)	0.656
New pacemaker implantation	1 (1.4)	1 (2.7)	1.000
New-onset atrial fibrillation	6 (8.7)	6 (16.2)	0.332
Left bundle branch block	1 (1.4)	0	1.000
Mechanical assisting device			**0.087**
IABP	5(7.2)	3 (8.1)	
ECMO	0	2 (5.4)	
IABP + ECMO	0	1 (2.7)	
30-day re-hospitalization	0	1 (2.7)	0.206
30-day mortality	15 (21.7)	2 (5.4)	0.057

*Values are mean ± SD, median (interquartile range), or n (%). IABP, intra-aortic balloon pump; ECMO, extracorporeal membrane oxygenation. The bold values refer p-values <0,05 (statistically significant)*.

In our sample, five cases have required further surgery: (1) exploratory thoracotomy due to hemothorax because of a laceration of an intercostal vein; (2 and 3) left ventricle laceration; (4) hemostasis revision; (5) hypertensive pneumothorax during central venous puncture. One patient required a second valve implantation due to migration of the prosthesis into the left atrium (without embolization). There seems to be no relationship between complications and valve disease etiology, however the number of patients who required new interventions is quite small and does not allow us from drawing firm conclusions in that regard. Clinical, laboratory, echocardiographic data and 30-day outcomes of patients that underwent mitral ViV procedure are shown in the Supplementary Table 4. In these patients, there was a higher incidence of baseline atrial fibrillation (88.9 vs. 58.8%; *p* = 0.008), and a higher number of previous surgeries in the rheumatic group (*p* < 0.001). In the analysis excluding tricuspid cases (Table 4), we found several differences between the two groups (rheumatic vs. non-rheumatic) related to age, sex, atrial fibrillation, creatinine, LVED diameter, LVEF, mechanical assisting device and number of previous surgeries (all with *p* < 0.05).

**Table 4:** Baseline clinical, laboratory, echocardiographic data and 30-day outcomes of patients undergoing mitral or aortic valve-in-valve procedure (excluding tricuspid valve-in-valve procedure).

	**Rheumatic (*N* = 69)**	**Non-rheumatic(*N* = 27)**	***P* value**
**Clinical data**			
Age, years	65 (57–70)	72 (65–81)	**0.023**
Body surface area, m^2^	1.6 (1.5–1.7)	1.7 (1.6–1.8)	0.069
Female sex	51 (73.9)	12 (44.4)	**0.013**
Diabetes	10 (14.5)	7 (25.9)	0.236
Hypertension	36 (52.2)	20 (74.1)	0.084
Poor mobility	16 (23.2)	5 (18.5)	0.823
Atrial fibrillation	57 (82.6)	13 (48.1)	**0.002**
Coronary artery disease	14 (20.3)	8 (29.6)	0.478
Previous heart failure hospitalization	14 (20.3)	5 (18.5)	1.000
Total of previous surgeries			** <0.001**
1	22 (31.9)	23 (85.2)	
2	22 (31.9)	3(11.1)	
3	19 (27.5)	1 (3.7)	
4	4 (5.8)	-	
5	1 (1.4)	-	
6	1 (1.4)	-	
STS-PROM score, %	6.61 (3.6–11.0)	5.64 (3.9-10.5)	1.000
**Symptoms**			
NYHA class			0.676
I	4 (5.8)	2 (7.4)	
II	18 (26.1)	4 (14.8)	
III	30 (43.5)	13 (48.1)	
IV	17 (24.6)	8 (29.6)	
Angina	9 (13)	3 (11.1)	1.000
**Laboratory**			
Hemoglobin, mg/dl	12.3 (10.4–13.5)	11.8 (11.0–13.4)	0.744
Creatinine, mg/dl	1.11 (0.9–1.3)	1.32 (1.0–1.5)	**0.023**
**Baseline echocardiography**			
LVED diameter, mm	50 (45.0–54.2)	57 (48–63)	**0.033**
LVES diameter, mm	33 (29.5–36.0)	39 (29.5–44.5)	0.06
LVED volume, ml	113 (88.0–132.2)	132.5 (94.5-167.0)	0.797
LVES volume, ml	42 (31–54)	60 (32.7–70.0)	0.139
LVEF, %	61 (56.2–66.0)	55 (45–63)	**0.037**
PSAP, mmHg	59.5 (45.5–73.5)	55 (45–63)	0.364
**Procedure data**			
Device success^*^	52 (75.4)	23 (85.2)	0.440
Pre-dilatation	1 (1.4)	3 (11.1)	0.066
Post-dilatation	23 (33.3)	6 (22.2)	0.413
Access			0.558
Transapical	63 (92.6)	26 (96.3)	
Jugular	1 (1.5)	-	
Transfemoral	2 (2.9)	1 (3.7)	
Transeptal	2 (2.9)	-	
Length of in-hospital stay, days	18 (10–30)	16 (8–25)	0.514
Outcomes			
Procedure mortality	1 (1.4)	1 (3.7)	0.486
In-hospital myocardial infarction	4 (5.8)	1 (3.7)	1.000
Major vascular complication	3 (4.3)	-	0.557
Major bleeding	7 (10.1)	4 (14.8)	0.497
Red blood cells transfusion	11 (15.9)	4 (14.8)	1.000
Acute kidney injury	10 (14.5)	6 (22.2)	0.373
Sepsis	19 (27.5)	6 (22.2)	0.783
Valve-related dysfunction requiring second valve implantation	-	1(3.7)	0.281
Valve-related dysfunction requiring valve surgery	4 (5.8)	1 (3.7)	1.000
New pacemaker implantation	1 (1.4)	1 (3.7)	0.486
New-onset atrial fibrillation	6 (8.7)	6 (23.1)	0.083
Left bundle branch block	1 (1.4)	-	1.000
Mechanical assisting device			**0.038**
IABP	5 (7.2)	3 (11.1)	
ECMO	-	2 (7.4)	
30-day re-hospitalization	3 (5.6)	4 (22.2)	0.061
30-day mortality	15 (21.5)	2 (7.4)	0.139

*Values are n (%) or median [IQR]*.

* *According to the modified Mitral Valve Academic Research Consortium (MVARC-2) without hemodynamic criteria and the Aortic Valve Academic Research Consortium-2 (VARC-2) (9, 10, 18, 19). ECMO, extracorporeal membrane oxygenation; eGFR, estimated glomerular filtration; IABP, intra-aortic balloon pump; LVED, Left ventricular end-diastolic; LVEF, left ventricular ejection fraction; LVES, Left ventricular end-systolic; NYHA, New York Heart Association; PSAP, pulmonary systolic arterial pressure; STS-PROM, Society of Thoracic Surgeons predicted risk of mortality. Bold values denote statistical significance*.

## Discussion

The main findings of this initial series, comparing transcatheter ViV procedures in patients with rheumatic vs. non-rheumatic severe bioprosthetic valve disfunction, were that although RHD patients presented a higher risk profile that included higher rates of female sex, atrial fibrillation and pulmonary hypertension, procedural success rates were similar between rheumatic vs. non-rheumatic patients. Also, despite a trend toward higher rates of mortality in the short-term, over a median follow-up of 20 months rheumatic etiology was not associated with increased mortality.

RHD is the main cause of acquired heart disease and cardiovascular mortality in young people worldwide. It is a condition of global importance as it is estimated ~36 million affected patients, ensuing in ~250,000 deaths per year, most often in underserved populations (20–22). These patients undergo cardiac surgery at younger age and are more frequently women with a higher burden of comorbidities (1). This is also the case of our study, so that RHD patients were ~2-fold more frequently women with atrial fibrillation, alongside a 10 mmHg higher mean PSAP. Furthermore, such rheumatic patients are frequently selected to a biological rather than mechanical valve, due to sociocultural context, comprising difficulties in keeping adequate warfarin control and risks of complications associated with the mechanical prosthesis (1, 7, 22). In developing countries, warfarin anticoagulation presents many logistic difficulties, including lack of facilities in close proximity to monitor the international normalized ratio (INR), employment activities with a greater risk of trauma and the large number of females in child-bearing age who become pregnant (23). These difficulties with anticoagulation, together with the more widespread availability of transcatheter valves, have encouraged in the last years the use of bioprosthetic valves by >70%, including new surgical valves with improved anticalcification treatment (24). Nonetheless, the use of bioprosthetic valves in rheumatic patients have a lower long-term performance and durability compared to other valve disease etiologies (25, 26). Thus, rheumatic patients often present with multiple previous open-heart surgeries, due to the natural biological valve prosthesis degeneration, also with higher rates (~20%) of simultaneous severe multivalvular disease requiring intervention (7, 27, 28).

In our study, the median number of prior surgical procedures was 2-fold higher in the RHD patients, so that 68.1% of them had ≥2 prior surgeries vs. 32.4% of non-rheumatic. Collectively such factors, including higher risk profile, together with multiple prior surgical procedures, can magnify the risks of morbidity and mortality up to ~2–3-fold (26–29). Not surprinsingly, despite similar rates of device success between rheumatic vs. non-rheumatic patients, mortality at the short-term was somewhat higher in RHD patients (21.7 vs. 5.4% in rheumatic vs. non-rheumatic patients, respectively). These relatively high mortality rates are similar to prior literature, where 30-day and 1 year mortality rates in mitral and aortic ViV procedures were ~8 and ~20%, respectively, although especific data in RHD patients undergoin transcatheter procedures are lacking (6, 19, 29, 30).

Furthermore, mortality risk for reoperation in patients with degenerated bioprosthesis ranges from 1.5 up to 23% (31). Valve surgery mortality increases proportionally to the number of previous operations, reaching the prohibitive value of 40% in the fourth mitral valve replacement (7, 32). Recent meta-analysis showed that ViV is associated with lower rate of MACE, bleeding and short hospitalization complications when compared to re-do surgery, being a reasonable treatment option (33). Besides, ViV procedure does not contraindicate further surgical or transcatheter procedures in the future, which is one of the advantages of transcatheter procedure. Nonetheless, it is important to consider several challenges when a surgical procedure is foreseen after a transcatheter procedure. For instance, in the context of aortic position several challenges may be encountered regarding the smaller annular size, possibly associating with worse hemodynamics in the future in case of a TAVI-in-TAVI, in addition to coronary access and eventually coronary obstruction. These issues have been evaluated recently in some registries worldwide (13), but data is lacking on ViV in patients with RHD vs. those with non-rheumatic etiology.

Of note, despite the higher short-term mortality seen in our study, at a median follow-up of 20 months rheumatic etiology was not significantly associated with longer term mortality. Most of our patients were treated using the transapical approach, that is known to jeopardize transcatheter outcomes, as compared to transfemoral and transeptal approaches (34). Therefore, the relatively low number of events at 30-days and in the follow-up precluded us from drawing firm conclusions on whether the rheumatic factor itself or the higher burden of comorbidities are resposible for such relatively higher mortality rates in the rheumatic patients, and this will have to be the scope of future larger studies. Likewise, future studies evaluating the different approaches and potential for transfemoral and transeptal approaches in improving clinical outcomes should also be evaluated in the near future.

## Limitations

This is a single-center, non-randomized data-base analyses, that despite the prospective data collection, has the limitations associated with the study design. For instance, there was no data available regarding ViV procedure duration in each group and post-procedure hemodynamic data (such as prosthesis-patient mismatch, gradients, and PVL) were evaluated only by echocardiography and not invasively. The number of patients was relatively small, albeit large for this clinical entity and procedure, with limited number of events that precluded the performance of a multivariable analysis. Therefore, the differences in baseline characteristics, such as the higher prevalence of pulmonary hypertension and atrial fibrillation in rheumatic group, may have influenced the distinct 30-day mortality rates, besides the lack of association in the long-term mortality in the univariable analysis. However, it is important to emphasize that our study is a real-world registry that represent the current practices. Learning curve may have also played a role in the different outcomes, as shown in our series of the first 50 cases undergoing mitral ViV procedures (7).

Another important point is the lack of data on transcatheter valve durability in rheumatic patients undergoing ViV procedure and given our median follow-up of 20 months merit additional evaluation in future larger studies with longer-term follow-up. Of note, recent studies in the field have shown good valve durability up to 8-years in aortic ViV procedures (35) and up to 4-years in mitral procedures (19).

## Conclusion

In conclusion, transcatheter ViV implantation is an acceptable alternative to redo operations in the treatment of patients with RHD and severe bioprosthetic valve dysfunction. Despite similar device success rates, rheumatic patients present higher 30-day mortality rates with good mid-term clinical outcomes. Future studies with a larger number of patients and follow-up will have to conclude on the role of transcatheter ViV procedures in RHD population.

## Data Availability Statement

The original contributions presented in the study are included in the article/Supplementary Material, further inquiries can be directed to the corresponding author/s.

## Ethics Statement

The studies involving human participants were reviewed and approved by Comissão de Ética para Análise de Projetos de Pesquisa—CAPPesq. The patients/participants provided their written informed consent to participate in this study.

## Author Contributions

ML, VR, and HR were the main responsible for the analysis of the data and paper writing. JP, AA, HR, MV, and FB performed the valve-in-valve procedures. JF, AS, RF, MS, and GS were responsible for data collection in medical records. FT, RS, and HR were responsible for the idealization of the study. All authors contributed to the article and approved the submitted version.

## Conflict of Interest

The authors declare that the research was conducted in the absence of any commercial or financial relationships that could be construed as a potential conflict of interest.

## Publisher's Note

All claims expressed in this article are solely those of the authors and do not necessarily represent those of their affiliated organizations, or those of the publisher, the editors and the reviewers. Any product that may be evaluated in this article, or claim that may be made by its manufacturer, is not guaranteed or endorsed by the publisher.
