# Supplementary material for: Transcatheter Valve-in-Valve Procedures for Bioprosthetic Valve Dysfunction in Patients With Rheumatic vs. Non-Rheumatic Valvular Heart Disease
Source: Front Cardiovasc Med. 2021 Aug 4;8:694339. doi: 10.3389/fcvm.2021.694339 (PMC8373457; doi:10.3389/fcvm.2021.694339)
Supplement: Supplementary file 1 [file Data_Sheet_1.docx]

**Supplemental Table 1.** Univariable analysis for modified device success*

|  | OR | 95% CI | | p |
| --- | --- | --- | --- | --- |
|  |  | Lower | Upper |  |
| Age, years | 0.993 | 0.962 | 1.024 | 0.648 |
| Body surface area, m² | 1.309 | 0.072 | 23.916 | 0.856 |
| Female sex | 0.630 | 0.222 | 1.788 | 0.385 |
| Diabetes | 1.183 | 0.307 | 4.564 | 0.807 |
| Hypertension | 1.427 | 0.548 | 3.718 | 0.467 |
| Poor mobility | 0.800 | 0.257 | 2.492 | 0.700 |
| Atrial fibrillation | 1.200 | 0.433 | 3.329 | 0.726 |
| Coronary artery disease | 0.800 | 0.257 | 2.492 | 0.700 |
| Previous heart failure hospitalization | 1.000 | 0.325 | 3.073 | 1.000 |
| Stroke and transient stroke | 0.986 | 0.292 | 3.329 | 0.981 |
| STS score, % | 0.969 | 0.888 | 1.057 | 0.479 |
| EuroSCORE II, % | 0.996 | 0.920 | 1.078 | 0.917 |
| Rheumatic etiology | 0.371 | 0.115 | 1.199 | 0.097 |
| Angina | 1.267 | 0.256 | 6.270 | 0.772 |
| Heart Failure | 0.820 | 0.282 | 2.385 | 0.716 |
| Hemoglobin, mg/dl | 1.081 | 0.853 | 1.370 | 0.521 |
| Creatinine, mg/dl | 0.791 | 0.331 | 1.888 | 0.597 |
| Left ventricular end-diastolic diameter, mm | 1.036 | 0.967 | 1.108 | 0.314 |
| Left ventricular end-systolic diameter, mm | 1.037 | 0.964 | 1.116 | 0.328 |
| Left ventricular end-diastolic volume, ml | 1.005 | 0.992 | 1.018 | 0.445 |
| Left ventricular end-systolic volume, ml | 1.009 | 0.990 | 1.029 | 0.360 |
| Left ventricular mass index g/m | 1.014 | 0.997 | 1.030 | 0.099 |
| Left ventricular ejection fraction, % | 0.995 | 0.949 | 1.044 | 0.839 |
| Pulmonary artery systolic pressure, mmHg | 0.997 | 0.972 | 1.023 | 0.837 |
| Transapical Access | 0.216 | 0.027 | 1.727 | 0.148 |
| Prosthesis position |  |  |  | 0.720 |
| Mitral | 2.291 | 0.475 | 11.059 | 0.302 |
| Aortic | - | - |  | 1.000 |
| Tricuspid | 1.964 | 0.221 | 17.421 | 0.545 |
| Number of previous surgeries | 1.562 | 0.924 | 2.641 | 0.096 |

*Device success for mitral procedures: absence of (i) procedural death, (ii) malposition/embolization/migration, (iii) second transcatheter heart valve, (iiii) left ventricular outflow tract obstruction and (iiiii) stroke. For the aortic procedures: (i) absence of procedural mortality, (ii) correct positioning of a single prosthetic heart valve into the proper anatomical location, (iii) no prosthesis–patient mismatch, (iiii) mean aortic valve gradient <20 mmHg and (iiiii) no moderate or severe prosthetic valve regurgitation. For the tricuspid procedures: (i) absence of reintervention, endocarditis or valve thrombus, (ii) absence of moderate or severe regurgitation and (iii) absence of mean gradient ≥10 mmHg.

**Supplemental Table 2.** Univariable analysis of 30-day predictors of mortality

|  | OR | 95% IC | | p |
| --- | --- | --- | --- | --- |
|  |  | Lower | Upper |  |
| Age, years | 0.991 | 0.960 | 1.022 | 0.551 |
| Body surface area, m² | 1.320 | 0.055 | 31.755 | 0.864 |
| Female sex | 1.484 | 0.480 | 4.581 | 0.493 |
| Diabetes | 0.658 | 0.136 | 3.182 | 0.602 |
| Hypertension | 0.522 | 0.182 | 1.495 | 0.226 |
| Poor mobility | 0.789 | 0.205 | 3.033 | 0.731 |
| Atrial fibrillation | 0.760 | 0.347 | 1.664 | 0.492 |
| Coronary artery disease | 0.789 | 0.205 | 3.033 | 0.731 |
| Chronic obstructive pulmonary disease | 1.679 | 0.662 | 4.263 | 0.275 |
| Peripheral arterial disease | - | - | - | 0.999 |
| Previous heart failure hospitalization | 0.982 | 0.289 | 3.336 | 0.976 |
| Stroke and transient stroke | 0.230 | 0.029 | 1.849 | 0.167 |
| STS score, % | 1.019 | 0.925 | 1.123 | 0.699 |
| EuroSCORE II, % | 0.934 | 0.836 | 1.045 | 0.232 |
| Rheumatic etiology | 4.861 | 1.047 | 22.573 | **0.044** |
| Angina | 1.053 | 0.209 | 5.298 | 0.950 |
| Hemoglobin, mg/dl | 0.917 | 0.709 | 1.185 | 0.506 |
| Creatinine, mg/dl | 1.238 | 0.483 | 3.176 | 0.657 |
| Platelets, cells/uL | 1.000 | 1.000 | 1.000 | 0.319 |
| Left ventricular ejection fraction, % | 0.993 | 0.945 | 1.043 | 0.776 |
| Pulmonary artery systolic pressure, mmHg | 0.999 | 0.971 | 1.027 | 0.938 |
| Transapical access | 3.507 | 0.433 | 28.394 | 0.240 |
| Prosthesis position |  |  |  |  |
| Mitral | 1.552 | 0.173 | 13.890 | 0.694 |
| Aortic | 0.400 | 0.021 | 7.484 | 0.540 |
| Tricuspid | - | - | - | 1.000 |
| Device success | 1.004 | 0.295 | 3.409 | 0.995 |
| Pre-dilatation | 0.467 | 0.124 | 1.756 | 0.260 |
| Post-dilatation | 0.414 | 0.110 | 1.555 | 0.192 |

Bold values denote statistical significance.

**Supplemental Table 3** - Post- procedure echocardiographic parameters

|  | | **Rheumatic** | | **Non-rheumatic** | | **P value** |
| --- | --- | --- | --- | --- | --- | --- |
| **Aortic Valve-in-Valve** Implantation | | **(N=12)** | | **(N=7)** | |  |
| Paravalvular Aortic Leak | |  | |  | | 0.351 |
| Trace | | 10 (83.3) | | 5 (71.4) | |  |
| Mild | | 1 (8.3) | | 2 (28.6) | |  |
| Mean Transaortic Gradient, mmHg | | 17 (11-23) | | 12 (10-26) | | 0.266 |
| Aortic Mismatch | | - | | 1 | | 1.000 |
| **Mitral Valve-in-Valve Implantation** | | **(N=61)** | | **(N=15)** | |  |
| Paravalvular Mitral Leak | |  | |  | | 0.175 |
| Trace | | 41 (67.2) | | 9 (60) | |  |
| Mild | | 10 (16.4) | | 3 (20) | |  |
| Moderate | | 1 (1.6) | | 2 (13.3) | |  |
| Mean Transmitral Gradient, mmHg | | 5 (4-6) | | 4 (4-5) | | 0.245 |
| Mitral Mismatch | | - | | - | | 1.000 |
|  |  | |  | |  |  |

Values are n (%) or median [IQR].

**Supplemental Table 4**: Baseline clinical, laboratory, echocardiographic data and 30-day outcomes of patients undergoing mitral valve-in-valve procedure.

|  | **Rheumatic**  **(N=63)** | **Non-rheumatic**  **(N=17)** | **P value** | |
| --- | --- | --- | --- | --- |
| **Clinical data** |  |  |  | |
| Age, years | 65.36 (54.7-70.5) | 69.63 (60.3-73.8) | 0.274 | |
| Body surface area, m² | 1.60 (1.50-1.68) | 1.70 (1.58-1.81) | 0.226 | |
| Female sex | 76.2 (48) | 52.9 (9) | 0.075 | |
| Diabetes | 8 (12.7) | 4 (23.5) | 0.271 | |
| Hypertension | 31 (49.2) | 11 (64.7) | 0.389 | |
| Poor mobility | 15 (23.8) | 3 (17.6) | 0.749 | |
| Atrial fibrillation | 56 (88.9) | 10 (58.8) | **0.008** | |
| Coronary artery disease | 10 (15.9) | 1 (5.9) | 0.441 | |
| Previous heart failure hospitalization | 10 (15.9) | 2 (11.8) | 1.000 | |
| Total of previous surgeries |  |  | **<0.001** | |
| 1 | 17 (27) | 15 (88.2) |  | |
| 2 | 22 (34.9) | 2 (11.8) |  | |
| 3 | 18 (28.6) | - |  | |
| 4 | 4 (6.3) | - |  | |
| 5 | 1 (1.6) | - |  | |
| 6 | 1 (1.6) | - |  | |
| STS-PROM score, % | 6.61 (3.96-11.16) | 6.64 (3.73-10.06) | 1.000 | |
| **Symptoms** |  |  |  | |
| NYHA class |  |  | 0.526 |  |
| I | 4 (6.3) | 1 (5.9) |  |  |
| II | 17 (27) | 2 (11.8) |  |  |
| III | 27 (42.9) | 10 (58.8) |  |  |
| IV | 15 (23.8) | 4 (23.5) |  |  |
| Angina | 8 (12.7) | 2 (11.8) | 1.000 | |
| **Laboratory** |  |  |  | |
| Hemoglobin, mg/dl | 12.2 (10.5-12.8) | 11.2 (10.0-12.9) | 0.585 | |
| Creatinine, mg/dl | 1.11 (0.88-1.37) | 1.24 (1.01-1.65) | 0.226 | |
| **Baseline echocardiography** |  |  |  | |
| LVED diameter, mm | 49 (45-52) | 55 (44-63) | 0.148 | |
| LVES diameter, mm | 32 (29-35) | 38 (28-42) | 0.079 | |
| LVED volume, ml | 108 (87-130) | 141 (88-167) | 0.41 | |
| LVES volume, ml | 41 (30-51) | 58 (30-70) | 0.221 | |
| LVEF, % | 62 (58-66) | 60 (53-65) | 0.358 | |
| PSAP, mmHg | 60 (46-74) | 58 (49-68) | 0.469 | |
| **Procedure data** |  |  |  | |
| Device success* | 46 (73) | 15 (88.2) | 0.335 | |
| Pre-dilatation | 1 (1.6) | - | 1.000 | |
| Post-dilatation | 19 (30.2) | 2 (11.8) | 0.230 | |
| Access |  |  |  | |
| Transapical | 58 (93.5) | 17 (100) |  | |
| Jugular | 1 (1.6) | - |  | |
| Transfemoral | 1 (1.6) | - |  | |
| Transeptal | 2 (3.2) | - |  | |
| Length of in-hospital stay, days | 17 (10-30) | 15 (8-28) | 0.667 | |
| **Outcomes** |  |  |  | |
| Procedure mortality | 1 (1.6) | 1 (5.9) | 0.382 | |
| In-hospital myocardial infarction | 3 (4.8) | - | 1.000 | |
| Major vascular complication | 3 (4.8) | - | 1.000 | |
| Major bleeding | 6 (9.5) | 1 (5.9) | 1.000 | |
| Red blood cells transfusion | 10 (15.9) | 2 (11.8) | 1.000 | |
| Acute kidney injury | 8 (12.7) | 3 (17.6) | 0.693 | |
| Sepsis | 16 (25.4) | 3 (17.6) | 0.749 | |
| Valve-related dysfunction requiring second valve implantation | - | 1(5.9) | 0.212 | |
| Valve-related dysfunction requiring valve surgery | 4 (6.3) | - | 0.573 | |
| New pacemaker implantation | - | 1 (5.9) | 0.212 | |
| New-onset atrial fibrillation | 6 (9.6) | 5 (31.3) | **0.04** | |
| Left bundle branch block | 1(1.6) | - | 1.000 | |
| Mechanical assisting device |  |  | 0.202 | |
| IABP | 5 (7.9) | 1 (5.9) |  | |
| ECMO | - | 1 (5.9) |  | |
| 30-day re-hospitalization | 3 (6.1) | 3 (21.4) | 0.118 | |
| 30-day mortality | 15 (23.8) | 1 (5.9) | 0.170 | |

Values are n (%) or median [IQR]. *According to the modified Mitral Valve Academic Research Consortium (MVARC-2) without hemodynamic criteria and the Aortic Valve Academic Research Consortium-2 (VARC-2) (10,11,19,20). ECMO: extracorporeal membrane oxygenation; eGFR: estimated glomerular filtration; IABP: intra-aortic balloon pump; LVED: Left ventricular end-diastolic; LVEF: left ventricular ejection fraction; LVES: Left ventricular end-systolic; NYHA: New York Heart Association; PSAP: pulmonary systolic arterial pressure; STS-PROM: Society of Thoracic Surgeons predicted risk of mortality. Bold values denote statistical significance.
